# Twelve Practical Tips for Integrating AI Into Medical Education: Tutorial to Support Educators Across Teaching, Research, Administration, and Ethical Domains

**DOI:** 10.2196/81297

**Published:** 2025-12-12

**Authors:** Alireza Jalali, Kadidja Harbi Houssein, Salomon Fotsing

**Affiliations:** 1 Faculty of Medicine University of Ottawa Ottawa, ON Canada

**Keywords:** artificial intelligence, ethics, educational innovation, AI literacy, medical education, educational technology

## Abstract

Artificial intelligence (AI) is rapidly reshaping medical education, offering new opportunities to personalize learning, enhance research, and streamline administration. The aim of this study is to provide 12 practical, evidence-informed tips by drawing on current literature and real-world examples to guide the integration of AI into medical education, supporting educators across teaching, research, administration, and ethical domains. Key strategies include using adaptive learning platforms to tailor educational content, using AI tools to provide timely feedback, and incorporating AI-generated clinical scenarios in case-based learning. The importance of fostering AI literacy among students is emphasized, as well as utilizing AI-powered tools for efficient literature reviews, data analysis, and manuscript preparation. Administrative applications such as automating routine tasks, supporting strategic planning through data analysis, and enhancing faculty development with AI-driven platforms are also discussed. Ethical considerations are highlighted, with a focus on ensuring transparency, fairness, and accountability in all AI applications. By following these 12 tips, medical educators can leverage the benefits of AI to improve educational outcomes, increase efficiency, and prepare future clinicians for a technology-driven health care environment.

## Introduction

Artificial intelligence (AI) is rapidly transforming medical education. It presents new opportunities for personalized learning, enhanced feedback, and streamlined administrative processes. Its impact is clear across multiple areas in medical schools, including medical student teaching, research, administrative governance, leadership paradigms, and ethical considerations.

In teaching, AI facilitates adaptive educational tools, intelligent clinical simulators, and automated assessment platforms. These technologies allow greater personalization of learning, helping students acquire clinical competencies in realistic simulated environments. Illustrative examples include intelligent tutoring systems and virtual patient simulators, which permit students to engage in risk-free clinical decision-making. Nevertheless, the integration of these tools into established curricula and the requisite faculty training present notable pedagogical challenges [1,2].

In the research field, AI serves as a catalytic agent in research by expediting the analysis of extensive biomedical datasets, accelerating the discovery of novel therapeutic interventions, and advancing personalized medicine. University researchers employ machine learning algorithms to discern intricate correlations within genomic, clinical, and imaging data. AI also automates labor-intensive tasks such as literature reviews and cohort selection, thereby allocating more time for critical analysis and experimental design. These advancements are substantiated by extensive work from institutions like Inserm (French National Institute of Health and Medical Research) and in research pertaining to health data integration [3,4].

On the administrative and leadership front, AI is redefining governance practices within medical education institutions. Its applications include optimizing human resource management, scheduling academic timetables, forecasting training requirements, and supporting strategic decision-making. Academic leaders are thus compelled to cultivate enhanced digital literacy to effectively oversee the ethical and efficient implementation of these technologies. The Quebec AI in Health Master Plan provides a pertinent example, outlining a strategic roadmap for AI integration across health and educational entities [5,6].

Finally, ethical considerations assume a central role in the integration of AI within medical education. Key issues encompass algorithmic transparency, the safeguarding of personal data for students and simulated patients, and the mitigation of potential biases within evaluation or diagnostic systems. These concerns underscore the critical need for reinforced ethical training for aspiring physicians, ensuring their responsible utilization of AI tools. Recent scholarship has identified over 70 ethical challenges associated with AI in education—many directly relevant to medical training [7,8]. Despite the substantial opportunities and expansive potential afforded by AI across these 4 sectors of medical education, a significant impediment persists: a considerable number of medical educators lack adequate training and resources for effective AI integration into their pedagogical practices. This deficiency is characterized by low digital literacy, a limited understanding of algorithmic principles, and a hesitancy to adopt tools perceived as complex or ethically ambiguous [7]. Furthermore, empirical studies indicate a pervasive sense of disempowerment among educators, attributable to the absence of specialized training and clear guidelines [2]. This paper will therefore delineate 12 practical recommendations designed to facilitate the integration of AI for the advancement of student instruction, research endeavors, administrative and leadership functions, and ethical considerations within medical education. Each recommendation appears to be informed by contemporary literature and best practices.

## Teaching

### Tip 1: Use AI to Personalize Learning Paths

Adaptive learning platforms powered by AI can tailor educational content to individual student needs, addressing unique learning requirements and competency rates. Sharma et al [9] highlight that adaptive learning is the “final piece of technology-enhanced learning” in medical education, demonstrating significant improvements in understanding and engagement. Commercial implementations such as Elsevier’s Cerego and McGraw Hill Education’s partnership exemplify the practical benefits of AI-driven personalization. Kellman’s [10] research on adaptive learning further supports this, showing educational improvements through repeated knowledge delivery and mastery criteria, with clinical trials in dermatology histopathology at University of California, Los Angeles, reporting significant pretest and posttest score improvements (*P*<.001).

### Tip 2: Enhance Feedback With AI Tools

Building on this personalization, AI can also strengthen another essential component of learning: feedback. AI tools provide timely, formative feedback during clinical simulations, fostering clinical judgment and reflective practice. Howell’s [11] study on AI-enhanced debriefing demonstrates that AI can generate real-time, personalized feedback and Socratic questions, leading to improved learner outcomes and knowledge retention. Practical applications such as Western Technical College’s AI chatbot Jennifer West loaded with open education resources and health care simulation standards illustrate how AI can support consistent, high-quality debrief sessions. Facilitators benefit by focusing on student interaction, while students report enhanced self-reflection and peer feedback.

### Tip 3: Integrate AI Into Case-Based Learning

Expanding on the role of AI in enhancing feedback, the next tip explores how AI can be integrated into case-based learning to further enrich clinical education. AI-generated clinical scenarios and virtual patients enrich problem-based learning by creating interactive, adaptive environments. The Diagnostic Reasoning and Its Development Group, Inc Group’s (2025) [12] research shows that AI-driven virtual patients can simulate real emotions, symptoms, and conditions, responding dynamically to student actions. This approach enhances clinical decision-making and interview skills in a safe, controlled setting. AI-powered tools prompt critical self-reflection such as asking, “What was your rationale for the administration of metoprolol?” or “How could holding the metoprolol change the patient’s outcome?”

### Tip 4: Train Students in AI Literacy

As AI becomes increasingly embedded in clinical education, it is equally important to ensure that students are equipped to use these tools responsibly. The next tip focuses on developing AI literacy to prepare learners for ethical and effective engagement with emerging technologies. Preparing students to critically evaluate and use AI tools is essential. The American Medical Association (2023) [13] emphasizes the importance of AI literacy in medical education, advocating for curricula that teach students to assess AI applications ethically and effectively. Although specific studies on AI literacy training are limited, the broader literature recognizes its critical role in preparing future clinicians for technology-driven health care environments.

## Research

### Tip 5: Accelerate Literature Reviews With AI

AI-powered summarization and search tools streamline systematic reviews by processing vast amounts of literature efficiently. Sharma et al [9] note that technology-enhanced learning has risen due to easy internet access, supporting the logical extension of AI tools for research. AI-powered platforms such as Perplexity.ai can quickly retrieve, summarize, and synthesize academic papers, accelerating the research process and helping summarize and synthesize relevant academic sources.

### Tip 6: Employ AI in Data Analysis

Beyond literature reviews, AI contributes to research through powerful tools for data analysis. Machine learning algorithms can analyze complex educational datasets, predict learner performance, and optimize content delivery [14]. The success of adaptive learning platforms in analyzing educational data patterns suggests similar benefits for research datasets in medical education [15].

### Tip 7: Collaborate With AI for Writing and Publishing

After supporting data analysis, AI can also help researchers with writing and publishing by assisting in drafting, editing, and formatting manuscripts. AI tools assist in drafting, editing, and formatting manuscripts, improving academic writing efficiency. Akbar’s [16] mixed methods study on doctoral students’ use of AI tools provides foundational evidence for AI’s role in academic research and writing processes. Researchers can use AI to brainstorm ideas, draft initial versions, and improve formatting but should ensure their final manuscript reflects their own critical thinking and contributions.

## Administration and Leadership

### Tip 8: Automate Routine Administrative Tasks

AI can automate scheduling, email management, and documentation, freeing faculty and staff for higher-value activities [17]. Although specific research on administrative automation in medical education is limited, the broader technology-enhanced learning literature supports its potential for efficiency gains [18].

### Tip 9: Use AI for Strategic Planning

Alongside simplifying daily administrative tasks, AI can guide strategic planning. By analyzing institutional data, it supports better decisions for curriculum design and resource use. AI can analyze institutional data to inform curriculum development and resource allocation. Bixler and Ceballos’s [19] conceptual model explores how AI supports educational leadership and decision-making, demonstrating its utility for strategic planning in medical education.

### Tip 10: Enhance Faculty Development With AI

In addition to guiding strategic planning, AI can also play a key role in supporting educators themselves. Through adaptive tools, it provides personalized learning and feedback to strengthen faculty development. AI-driven platforms can provide personalized learning and feedback for faculty, mirroring the benefits seen in student learning. The principles of adaptive learning apply equally to faculty professional development, enabling customized experiences and ongoing support [20].

## Ethical Considerations

### Tip 11: Uphold Ethical Use of AI

Transparency, fairness, and accountability must guide all AI applications in medical education. Weidener and Fischer’s [21] scoping review of AI ethics teaching in medical education highlights the importance of ethical instruction and practice. The American Medical Association (2023) [13] further reinforces the need for ethical standards, advocating for the responsible integration of AI. Additionally, educators should consider nuanced challenges such as the impact of AI-generated assessments or content creation on student autonomy and academic integrity, ensuring that AI supports rather than replaces critical thinking and professional development.

## Future

### Tip 12: Stay Informed and Adaptive

Maintaining ethical standards goes hand in hand with staying up-to-date on AI’s rapid developments. By continuously learning, educators can adapt responsibly and uphold best practices in medical education. The rapid evolution of AI in medical education necessitates continuous learning and adaptation. Sharma et al [9] emphasize that ongoing attention to emerging developments is essential, as the field is likely to change significantly over time.

## Practical Tips for Integrating AI Into Medical Education

Table 1 outlines 12 actionable strategies (Multimedia Appendix 1) for effectively integrating AI into medical education, with suggested tools and expected outcomes.

**Table 1:** Twelve practical tips for integrating AI^a^ into medical education.

Tip title	Description	Example tools/platforms	Key outcomes
Tip 1: Use AI to personalize learning paths	Adaptive platforms tailor content to individual student needs, improving engagement and mastery	Cerego Smart Sparrow McGraw Hill ALEKS	Improved learner engagement Personalized progression
Tip 2: Enhance feedback with AI tools	AI delivers immediate, personalized feedback in clinical simulations, promoting reflective learning	Jennifer West (AI chatbot) SimConverse FeedbackFruits	Accelerated skill development Deeper reflection
Tip 3: Integrate AI into case-based learning	AI generates dynamic clinical scenarios and virtual standardized patients for realistic decision-making practice	Diagnostic Reasoning and Its Development Group, Inc Clinician Body Interact Open-Source Clinical Application Resource	Improved diagnostic reasoning Clinical readiness
Tip 4: Train students in AI literacy	Teaching AI literacy prepares students to evaluate, interpret, and use AI tools ethically and effectively	Artificial Intelligence for Health (AI4HealthEd) American Medical Association AI Curriculum Google Teachable Machine	Greater digital competency Ethical awareness
Tip 5: Use AI to accelerate literature reviews	AI simplifies literature searches and synthesis, saving time and broadening evidence coverage	Perplexity.ai Elicit ResearchRabbit	Faster research preparation Improved evidence integration
Tip 6: Use AI for data analysis	AI analyzes complex datasets to predict performance and optimize learning strategies	RapidMiner Orange IBM SPSS Modeler	Data-driven decision-making Targeted interventions
Tip 7: Collaborate with AI for writing and publishing	AI assists in drafting, editing, and formatting manuscripts while ensuring responsible authorship and academic integrity	Grammarly ChatGPT Writefull	Enhanced writing quality Efficient publishing workflow
Tip 8: Automate routine administrative tasks	AI manages scheduling, email correspondence, and documentation to free educators’ time for strategic work	Explainable Artificial Intelligence (x.ai) Clara Google Duplex	Increased faculty productivity Streamlined operations
Tip 9: Use AI for strategic planning	AI analyzes institutional data to inform curriculum design, forecasting, and resource allocation	Tableau IBM Watson Power Business Intelligence	Informed planning Optimized resource management
Tip 10: Enhance faculty development with AI	AI platforms deliver adaptive learning and feedback for continuous professional growth	LinkedIn Learning Coursera AI Tracks Education Application (EdApp)	Ongoing educator improvement Personalized learning
Tip 11: Uphold ethical use of AI	Implement transparent, fair, and accountable AI practices in all academic and clinical settings	Ethics guidelines (American Medical Association, United Nations Educational, Scientific and Cultural Organization) Explainable AI tools	Trustworthy AI adoption Regulatory compliance
Tip 12: Stay informed and adaptive	Continuous learning helps educators keep pace with AI advancements and emerging best practices	AI newsletters PubMed alerts arXiv.org	Sustained innovation Up-to-date knowledge

^a^AI: artificial intelligence.

### Limitations

Although all 12 tips are grounded in published literature, they have not yet undergone external evaluation or been tested in educational practice. The paper synthesizes existing evidence but does not include longitudinal outcome data. Future studies could assess the practical impact and effectiveness of these recommendations in real-world settings.

### Conclusion

The integration of AI into medical education is supported by robust evidence, particularly in teaching applications, with emerging support in research, administrative, and ethical domains. AI enhances personalized learning, improves feedback quality, enriches case-based instruction, and supports institutional decision-making. However, maintaining human-centered approaches, ensuring ethical implementation, and continuously updating knowledge are critical. Future research should focus on long-term outcomes, comparative effectiveness, and expanded investigation of AI applications in administrative and research functions.

## Appendix

Multimedia Appendix 1 A visual poster of the 12 actionable strategies for effectively integrating artificial intelligence into medical education.

## Abbreviations

AI: artificial intelligence
