# Supplementary material for: Twelve Practical Tips for Integrating AI Into Medical Education: Tutorial to Support Educators Across Teaching, Research, Administration, and Ethical Domains
Source: JMIR Med Educ. 2025 Dec 12;11:e81297. doi: 10.2196/81297 (PMC12743235; doi:10.2196/81297)
Supplement: Multimedia Appendix 1 [file mededu_v11i1e81297_app1.pdf]

TWELVE PRACTICAL TIPS FOR INTEGRATING AI INTO MEDICAL EDUCATION

Alireza Jalali, MD, Kadidja Harbi, BSc (Hons), Salomon Fotsing MD, M.A. Ed
Faculty of Medicine, University of Ottawa
Francophone Affairs, Faculty of Medicine, University of Ottawa

INTRODUCTION

Artificial Intelligence (AI) is rapidly reshaping medical education, offering new opportunities to personalize learning, enhance research, and streamline administration. The aim of this article is to provide twelve practical, evidence-informed tips to guide the integration of AI into medical education, supporting educators across teaching, research, administration, and ethical domains. This article presents twelve practical tips for integrating AI into medical education, drawing on current literature and real-world examples. Key strategies include using adaptive learning platforms to tailor educational content, using AI tools to provide timely feedback, and incorporating AI-generated clinical scenarios in case-based learning

CATEGORY 1: ENHANCING LEARNER EXPERIENCE

Tip 1: Personalize Learning Paths

Key Outcomes: Improved learner engagement, personalized progression

Description: Use adaptive learning platforms to tailor educational content. AI adjusts difficulty and pacing based on individual performance to optimize learning outcomes

Example Tools: Cerego, Smart Sparrow, McGraw Hill ALEKS

Tip 2: Enhance Feedback

Key Outcomes: Accelerated skill development, deeper reflection

Description: Provide immediate, personalized feedback in simulations to improve clinical reasoning and decision-making skills through AI-powered analysis

Example Tools: Jennifer West (AI chatbot), SimConverse, FeedbackFruits

Tip 3: Integrate into Case-Based Learning

Key Outcomes: Improved diagnostic reasoning, clinical readiness

Description: Generate dynamic scenarios and virtual standardized patients for immersive case-based learning experiences that enhance clinical exposure

Example Tools: DxR Clinician, Body Interact, OSCAR

Tip 4: Train in AI Literacy

Key Outcomes: Greater digital competency, ethical awareness

Description: Prepare students to evaluate, interpret, and use AI ethically in clinical practice, fostering AI literacy among students to ensure they can effectively work in a technology-driven healthcare environment.

Example Tools: AI4HealthEd, AMA AI Curriculum, Google Teachable Machine

CATEGORY 2: IMPROVING RESEARCH & SCHOLARSHIP

Tip 5: Accelerate Literature Reviews

Key Outcomes: Faster research preparation, improved evidence integration

Description: Utilize AI-powered tools for efficient literature reviews, data analysis, and manuscript preparation to simplify searches and broaden evidence coverage for comprehensive research

Example Tools: Perplexity.ai, Elicit, Research Rabbit

Tip 6: Use for Data Analysis

Key Outcomes: Data-driven decision-making, targeted interventions

Description: Analyze complex datasets to predict performance and optimize educational strategies through pattern recognition and predictive analytics.

Example Tools: RapidMiner, Orange, IBM SPSS Modeler

Tip 7: Collaborate for Writing/Publishing

Key Outcomes: Enhanced writing quality, efficient publishing workflow

Description: Assist in drafting, editing, and ensuring responsible authorship while maintaining academic integrity and proper attribution.

Example Tools: Grammarly, ChatGPT, Writefull

CATEGORY 3: OPTIMIZING FACULTY & INSTITUTION

Tip 8: Automate Routine Admin Tasks

Key Outcomes: Increased faculty productivity, streamlined operations

Description: Manage scheduling and documentation to free educators' time for more meaningful teaching and mentoring activities through AI automation.

Example Tools: x.ai, Clara, Google Duplex

Tip 9: Use for Strategic Planning

Key Outcomes: Informed planning, optimized resource management

Description: Inform curriculum design, forecasting, and resource allocation through data analysis to support strategic planning and decision-making.

Example Tools: Tableau, IBM Watson, Power BI

Tip 10: Enhance Faculty Development

Key Outcomes: Ongoing educator improvement, personalized learning

Description: Deliver adaptive learning and feedback for continuous professional growth with AI-driven platforms that enhance faculty capabilities.

Example Tools: LinkedIn Learning, Coursera AI Tracks, EdApp

Tip 11: Uphold Ethical Use of AI

Key Outcomes: Trustworthy AI adoption, regulatory compliance

Description: Implement transparent and accountable AI practices ensuring transparency and accountability within medical education.

Example Tools: Ethics Guidelines (AMA, UNESCO), Explainable AI tools

Tip 12: Stay Informed and Adaptive

Key Outcomes: AI newsletters, PubMed alerts, arXiv.org

Description: Continuous learning to keep pace with advancements and best practices in AI technology to ensure effective and responsible implementation.

Example Tools: Sustained innovation, up-to-date knowledge

CONCLUSION

By following these twelve tips, medical educators can leverage the benefits of AI to improve educational outcomes, increase efficiency, and prepare future clinicians for a technology-driven healthcare environment. The importance of fostering AI literacy among students is emphasized, as well as utilizing AI-powered tools for efficient literature reviews, data analysis, and manuscript preparation. Administrative applications such as automating routine tasks, supporting strategic planning through data analysis, and enhancing faculty development with AI-driven platforms are also discussed

REFERENCES

CONTACT

Dr. Ali Jalali
ajalali@uottawa.ca
